# Mouse model phenotypes provide information about human drug targets

**DOI:** 10.1093/bioinformatics/btt613

**Published:** 2013-10-24

**Authors:** Robert Hoehndorf, Tanya Hiebert, Nigel W. Hardy, Paul N. Schofield, Georgios V. Gkoutos, Michel Dumontier

**Affiliations:** ^1^Department of Computer Science, University of Aberystwyth, Old College, King Street, Aberystwyth SY23 2AX, ^2^Department of Biology, Institute of Biochemistry and School of Computer Science, Carleton University, 1125 Colonel By Drive, Ottawa, Ontario K1S 5B6, Canada and ^3^Department of Physiology, Development and Neuroscience, University of Cambridge, Downing Street, Cambridge CB2 3EG, UK

## Abstract

**Motivation:** Methods for computational drug target identification use information from diverse information sources to predict or prioritize drug targets for known drugs. One set of resources that has been relatively neglected for drug repurposing is animal model phenotype.

**Results:** We investigate the use of mouse model phenotypes for drug target identification. To achieve this goal, we first integrate mouse model phenotypes and drug effects, and then systematically compare the phenotypic similarity between mouse models and drug effect profiles. We find a high similarity between phenotypes resulting from loss-of-function mutations and drug effects resulting from the inhibition of a protein through a drug action, and demonstrate how this approach can be used to suggest candidate drug targets.

**Availability and implementation:** Analysis code and supplementary data files are available on the project Web site at https://drugeffects.googlecode.com.

**Contact:** leechuck@leechuck.de or roh25@aber.ac.uk

**Supplementary information:** Supplementary data are available at *Bioinformatics* online.

## 1 INTRODUCTION

A major challenge currently faced by pharmacological research is the high rate of attrition in the development of new compounds, the increased cost of drug development and increased regulatory concern about drug safety and efficacy (Sleigh and Barton, 2010). As a result, pharmacological research is beginning to focus on *repurposing* existing drugs for new indications, and several large national and international research initiatives have begun to systematically address drug repurposing on a broad scale (Allison, 2012).

Strategies for drug repurposing can be divided into two main types: identification of *new targets* for known drugs and identification of *new indications* for a known mechanism of action (Sleigh and Barton, 2010). Approaches to drug repurposing include database-driven bioinformatics approaches, *in vivo* and *ex vivo* studies and high-throughput screening methods (Sleigh and Barton, 2010). Examples of computational approaches to drug repurposing include side effect-based approaches, in which similarity between drug effects is used to suggest drug targets and drug indications (Campillos *et al.*, 2008), data mining of clinical records (Tatonetti *et al.*, 2012) and approaches based on analysis of GWAS data (Sanseau *et al.*, 2012). Computational approaches to drug repurposing have the highest chance of succeeding if multiple independent data sources and analysis approaches are combined so that data from several independent domains and studies can be used to identify strong evidence for novel drug indications. Based on integrating multiple complementary datasets, integrative computational approaches can use multiple measures to prioritize candidate targets and drugs (Chen *et al.*, 2012b; Gottlieb *et al.*, 2011; Thorn *et al.*, 2010).

One set of resources that has been relatively neglected for drug repurposing is animal model phenotype (Hoehndorf *et al.*, 2012; Hurle *et al.*, 2013). The use of non-human species to investigate physiology and pathobiology, and the creation of animal models of human diseases amenable to experimental investigation, has become a successful paradigm in the biomedical sciences (Rosenthal and Brown, 2007). The development of high-throughput phenotyping has further increased the available amount of phenotype data resulting from targeted mutations in animal models, and pan-genomic projects such as the International Mouse Phenotyping Consortium (IMPC) (Brown and Moore, 2012) aim to delete every protein-coding gene in an organism and to identify the phenotypes resulting from these mutations. It has now become a challenge to systematically analyze the resulting data and use them to provide insights into human health and novel intervention strategies.

**Fig. 1.**
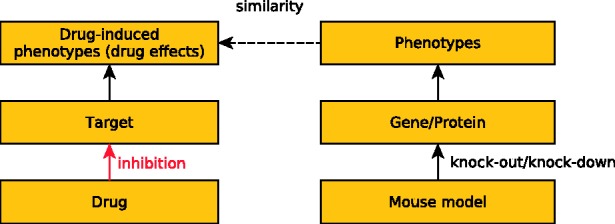
The figure illustrates our basic workflow and the connections between the different datasets we exploit. We aim to infer *inhibition* actions between drugs and their targets based on the similarity between drug effect profiles and mouse model phenotypes resulting from single gene knockouts. We test the hypothesis whether the phenotypic effects of a perturbation of a gene/protein through a drug action bears some similarity to the phenotypic effects of a targeted mutation of that gene/protein observed in a model organism. As drugs often perturb multiple genes/proteins, we systematically compute how well a drug effect profile covers observed phenotypes in a mouse model using a non-symmetrical measure of semantic similarity

In the past, several studies have used animal model data to suggest candidate genes for genetically based diseases (Chen *et al.*, 2012a; Hoehndorf *et al.*, 2011b), and one study also suggests that mouse model phenotypes can be used to provide insights into drug actions and drug effects in humans despite experimental differences between the two species (Kuhn *et al.*, 2013). Here, we use a phenome-wide approach to systematically compare drug effects with mutant mouse phenotypes (Fig. 1). We provide strong supporting evidence for the hypothesis that the similarity between drug effects and mouse phenotypes that result from loss of protein function indicates a similarity in the mechanism of action, i.e. an inhibition of the protein through the drug. We evaluate our results with experimentally validated lists of known drug targets and demonstrate on a genomic scale that a similarity between drug effects and mutant mouse phenotypes can reveal drug targets. Our approach opens the possibility for a systematic analysis of animal model phenotypes for candidate drug targets, and has a significant impact for integrative computational approaches to drug repurposing.

## 2 MATERIAL AND METHODS

### 2.1 Mouse model phenotypes

We use the Mammalian Phenotype (MP) Ontology (Smith *et al.*, 2004) and the Human Phenotype Ontology (HPO) (Robinson *et al.*, 2008), both downloaded on February 14, 2013 from the OBO Foundry Web site (http://obofoundry.org). We obtain the entity-quality definitions attached to MP and HPO from https://phenotype-ontologies.googlecode.com (downloaded on February 14, 2013).

We downloaded mouse phenotype data from the Mouse Genome Informatics (MGI) database (Blake *et al.*, 2011) on February 14, 2013. We obtained only mouse model phenotypes associated with models resulting from loss-of-function mutations in single genes.

### 2.2 Drug effect profiles and drug targets

Drug effect profiles were obtained from SIDER 2 (released on March 16, 2012) (Kuhn *et al.*, 2010). For each drug, we identify the STITCH identifier associated with the drug. We ignore all drugs for which no STITCH identifier has been identified in the SIDER dataset. STITCH identifiers are based on the STITCH database, version 3.1 (Kuhn *et al.*, 2012).

### 2.3 Integrating drug effects and phenotypes

We used a combination of lexical mapping, manual curation and exploitation of cross-references to map the Unified Medical Language System (UMLS) terms used to characterize SIDER’s drug profiles to the Human and Mammalian Phenotype Ontologies. Using exact lexical matching of UMLS terms to term names and synonyms in ontologies, we mapped 597 terms from SIDER to the HPO (Robinson *et al.*, 2008) and 262 terms from SIDER to the MP Ontology (Smith *et al.*, 2004). HPO already contains cross-references to terms from the UMLS (Bodenreider, 2004), 3858 of which can be found in SIDER’s drug effect profiles. We sorted the remaining SIDER terms for which we could not obtain a mapping to HPO or MP based on the frequency of their occurrence in SIDER drug effect profiles and manually mapped 953 of the most frequently occurring terms to HPO and 240 of the most frequently occurring terms to MP. The mappings are available on the project Web site.

### 2.4 Cross-species integration of phenotypes

Although we have annotated SIDER with both MP and HPO terms, mouse phenotypes are represented exclusively using MP. To make HPO and MP phenotype terms comparable, we use the PhenomeNET system of integrating phenotypes across species (Hoehndorf *et al.*, 2011b, 2013). PhenomeNET enables the *direct* comparison of phenotypes across multiple species (Hoehndorf *et al.*, 2011b, 2013), including mouse model phenotypes (describing using the MP) and human drug effects (described using the UMLS and mapped to HPO using our approach).

PhenomeNET uses an ontology-based integration framework that integrates phenotypes in different species based on species-independent ontologies and the PATO framework (Gkoutos *et al.*, 2005). In particular, PhenomeNET uses the large number of entity-quality-based definitions that have been created for species-specific phenotype ontology (Mungall *et al.*, 2010) and integrates them with species-independent ontologies. Entity-quality definitions of phenotypes decompose phenotype terms in an affected *entity* and a *quality* that characterizes how the entity is affected. For example, the phenotype term *proximal fibular overgrowth* (HP:0007126) is decomposed into the entity *proximal epiphysis of fibula* (FMA:33729) and the quality *hypertrophic* (PATO:0000584). Similarly, the mouse phenotype term *abnormal fibula morphology* (MP:0002187) is decomposed into the entity *fibula* (MA:0001360) and the quality *morphology* (PATO:0000051) with the qualifier *abnormal* (PATO:0000460).

Phenotypes in which *biological processes*, *functions* or *cellular components* are affected can then be integrated across species based on the Gene Ontology (GO) (Ashburner *et al.*, 2000), and phenotypes in which *anatomical structures* are affected are integrated based on homologous anatomical structures represented in the UBERON ontology (Mungall *et al.*, 2012). Using automated reasoning (Kazakov *et al.*, 2011), it then becomes possible to systematically identify equivalent, more specific and more general phenotypes across multiple species. For example, based on axioms in the combined ontology, we can infer that *proximal fibular overgrowth* is a more specific phenotype term than *abnormal fibula morphology* using the information that

- *Fibula* (MA:0001360) is **homologous to** *fibula* (FMA:24479) (from the UBERON ontology),
- *Proximal epiphysis of fibula* (FMA:33729) is a **part-of** *fibula* (FMA:24479) (from the FMA) and
- *Hypertrophic* (PATO:0000584) is a more specific quality than *morphology (abnormal)* (PATO:0000460).

Additional details for integrating phenotype ontologies across species using the Web Ontology Language (Grau *et al.*, 2008) are discussed in prior work (Hoehndorf *et al.*, 2010; 2011a, b).

### 2.5 Semantic similarity

Traditional semantic similarity measures are symmetrical, i.e. the similarity between *X* and *Y* is the same as the similarity between *Y* and *X*. As drugs may bind to multiple targets to elicit their effects (Kuhn *et al.*, 2013), we designed a novel non-symmetrical similarity measure based on the well-known SimGIC measure (Pesquita *et al.*, 2009). SimGIC is a group-based measure of semantic similarity, i.e. it compares two sets of annotations directly and is based on the Jaccard index weighted by the information content of ontology terms (Pesquita *et al.*, 2009).

We first select all phenotypes observed for single gene deletions in mice. For each gene *G* for which phenotype data are present in the MGI database, we then generate the union of the phenotypes observed in all models in which *G* has been deleted. The resulting phenotypes for a gene *G* are all phenotypes observed in mouse models in which *G* (and only *G*) has been deleted and provides a global view on the phenotypes associated with deletions of *G*.

We then add the super-classes of the phenotype annotations of each mouse model and drug to their set of annotations. In particular, if the HPO or MP phenotype *P* is a phenotype annotation associated with gene or drug *X*, and the super-classes of *X* in MP are the classes *Sup*(*X*), we add *Sup*(*X*) as annotations to *X*. To compute super-classes, we use the combined ontology of MP and HPO that forms part of PhenomeNET and enables cross-species comparisons of phenotypes (i.e. a class in MP may be a super-class of a class in HPO and vice versa) (Hoehndorf *et al.*, 2011b). We make the MP-based representation of drug effects in SIDER based on which we compute the similarity available on the project Web site.

We then define the information content *IC*(*t*) of an MP phenotype term *t* based on the probability 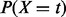 that a drug or mutant mouse model is characterized with *t*:
(1)
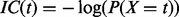

The probability 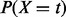 is empirically derived within the corpus of mouse models and drug profiles. We use only the structure of the MP to compute semantic similarity based on prior work that has shown that MP-based similarity measures outperform measures that use HPO or the combination of HPO and MP for analyzing mouse phenotype data (Oellrich *et al.*, 2012).

Given a drug effect profile *D* and a mutant mouse model *M*, where *D* is characterized by the ontology classes 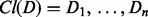 and *M* is characterized by the classes 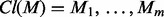, we define the similarity between *D* and *M* as:
(2)
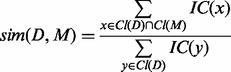

As a result, we obtain a similarity matrix between drug effect profiles and mouse model phenotypes (resulting from deletions of one gene). The similarity measure used is non-symmetrical and determines the amount of information about a drug effect profile *D* that is covered by a set of mouse model phenotypes *M*.

### 2.6 Evaluation datasets

Our approach is based on identifying a similarity between drug effect profiles and mouse model phenotypes. The STITCH database provides us with a set of drug–protein interactions in the mouse. We filter these interactions for those in which the mode of action is ‘inhibition’ (in the STITCH file actions.v3.1.tsv) and use this dataset directly as evaluation dataset ‘STITCH (mouse)’.

As we primarily aim to predict drug targets in human, we use the human–mouse orthology provided by the MGI database (Blake *et al.*, 2011) to obtain the mouse ortholog for each human gene that is a target of a STITCH compound, and use the mouse ortholog of the human drug target as a positive hit for the STITCH compound. We use the human drug–protein interactions provided by STITCH in which the mode of action is ‘inhibition’ as evaluation dataset ‘STITCH (human)’, and the human drug targets provided by DrugBank in which the mode of action is ‘inhibition’ as evaluation dataset ‘DrugBank’.

The STITCH database accumulates data from multiple sources and contains a confidence value for each interaction. The confidence ranges between 0 and 1, with an implicit cutoff value of 0.15. To evaluate the results of our analysis under different degrees of confidence, we generated evaluation datasets for STITCH in which we require a confidence of at least 0.5, and another dataset in which we require a confidence of at least 0.7. The evaluation datasets we used are available on the project Web site.

### 2.7 Receiver operating characteristic analysis and approximation of confidence intervals

To compute true- and false-positive rate, we iterate through the ranks of the generated similarity matrix (between drugs and mouse models) and compute, for each rank, the proportion of known drug targets in each of our evaluation datasets identified up to this rank (true-positive rate) as well as the proportion of targets not in the evaluation dataset included up to this rank (false-positive rate). We then use an analysis of the receiver operating characteristic (ROC) curve to evaluate and quantify the results. An ROC curve is a plot of the true-positive rate as a function of the false-positive rate and can be used to evaluate the performance of a classifier (Fawcett, 2006).

Confidence intervals for the area under the ROC curve (ROCAUC) are computed under the assumption of a normal distribution of ROCAUC values and using an estimate of the maximum variance of the ROCAUC as 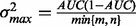, with *m* and *n* being the number of positive and negative instances in the evaluation dataset (Birnbaum and Klose, 1957). We then use 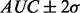 as an estimate of the 95% confidence interval (Cortes and Mohri, 2005).

## 3 RESULTS

### 3.1 Mouse model phenotypes provide information about drug targets

The hypothesis we test is whether a similarity between drug *D*’s effects and phenotypes resulting from *knock-out/knock-down* of a single gene (product) in an animal model can be used to indicate that *D inhibits* the gene (product) or its human ortholog, and whether phenotype similarity between mouse models and drug effects can be used to provide insights relevant for discovery of targets for known drugs. To test these hypotheses, we first made drug effects and mouse phenotypes comparable by mapping the drug effects described in the SIDER database (Kuhn *et al.*, 2010) with human and mouse phenotype terms, and then integrating human and mouse phenotypes across species (see Section 2).

Once mouse model phenotypes and human drug effects are made directly comparable, we use a measure of semantic similarity (Pesquita *et al.*, 2008) to compare drug effect profiles with mutant mouse phenotypes. We systematically compare the sets of phenotypes that have been observed in mice with single gene deletions with drug effect profiles obtained from the SIDER database, and use their similarity to prioritize candidate drug–protein interactions. To account for drugs’ binding to multiple targets, we developed a novel similarity measure between drug effect profiles and mouse model phenotypes that determines how much of the information in the drug effect profile can be explained through a set of mouse model phenotypes (Section 2). A schematic overview of the approach is shown in Figure 1.

We evaluate the results using three datasets: the human drug targets available in DrugBank (Knox *et al.*, 2011), the human drug targets available in the STITCH database (Kuhn *et al.*, 2012) and the mouse drug targets available in the STITCH database. DrugBank contains experimentally validated drug targets and includes information on the mode of action. Similarly, STITCH accumulates information about human and animal drug targets from multiple sources and includes the mode of action, if known. For our evaluation, we select only drug targets for which the mechanism of action is *inhibition*, as we aim to test whether these provide a similar phenotypic response as a knock-out/knock-down of the target.

**Fig. 2.**
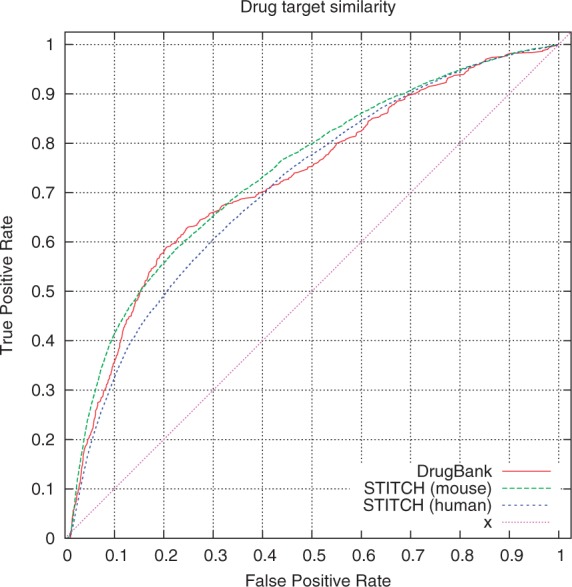
The ROC curves for our three evaluation datasets. DrugBank consists of experimentally verified and manually annotated drug-target interactions. STITCH integrates drug-target relations from multiple databases (including DrugBank), applies text mining and network-based inference approaches to infer drug-target relations. We used human–mouse orthology available from the MGI database to map human proteins in the DrugBank and STITCH (human) dataset to mouse proteins

For each drug, we identify the similarity between its pharmacological effects and the phenotypes observed in mouse models with a single gene deletion and rank the mouse models, for each drug, based on their similarity to the drug effect profile. We then evaluate the resulting ranks using positive instances of drug-target interactions, and Figure 2 shows the resulting ROC curves for the three main datasets we used. The ROCAUC values are 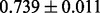 for mouse targets (STITCH), 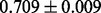 for human targets (STITCH) and 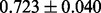 for human targets (DrugBank). We further restricted the STITCH evaluation datasets for human and mouse to high-confidence drug–protein interactions. With a confidence cutoff of 0.5, the resulting AUCs are 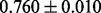 for human targets and 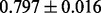 for mouse targets, and with a cutoff of 0.7, the AUCs are 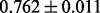 for human and 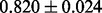 for mouse targets.

### 3.2 Targets in different protein families can be predicted with different accuracy

**Fig. 3.**
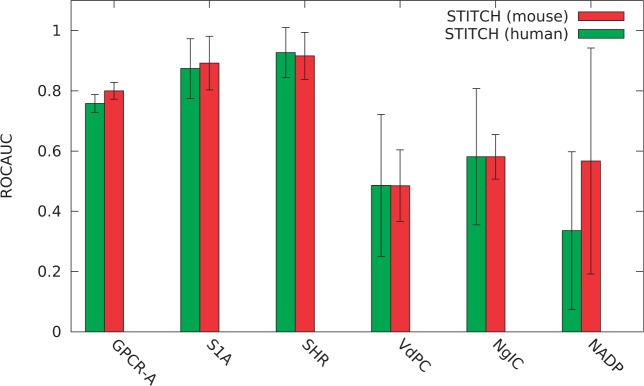
ROCAUC values obtained for STITCH (human) and STITCH (mouse) drug-target interactions grouped by protein family. *GPCR-A* stands for G protein-coupled receptor, rhodopsin-like (IPR000276), *S1A* for Peptidase S1A, chymotrypsin-type (IPR001314, *SHR* for Steroid hormone receptor (IPR001723), *VdPC* for voltage-dependent potassium channel (IPR003091), *NgIC* for Neurotransmitter-gated ion-channel (IPR006201) and NADP for NAD(P)-binding domain (IPR016040)

We further investigated whether our approach is more successful for particular protein families or particular drug categories. For this purpose, we performed our analysis for each of the top-level InterPro (Mulder *et al.*, 2005) protein families. To maintain statistical significance, we restrict our analysis to protein families in which we could identify >5 positive instances from our evaluation datasets, resulting in only six protein families for which we perform the analysis using the two STITCH-based evaluation datasets. The resulting ROCAUCs are shown in Table 1 and Figure 3. The ROC AUCs range from 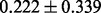 (for NAD(P)-binding domain proteins) to 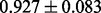 (for Steroid hormone receptor proteins).

**Table 1.** The ROCAUC values we obtain for different protein families, including the 95% confidence interval

InterPro family	ROCAUC (STITCH mouse)	ROCAUC (STITCH human)	ROCAUC (DrugBank)
G protein-coupled receptor, rhodopsin-like (IPR000276)	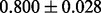	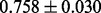	
Peptidase S1A, chymotrypsin-type (IPR001314)	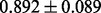	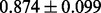	
Steroid hormone receptor (IPR001723)	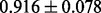	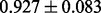	
Voltage-dependent potassium channel (IPR003091)	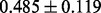	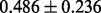	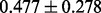
Neurotransmitter-gated ion-channel (IPR006201)	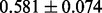	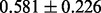	
NAD(P)-binding domain (IPR016040)	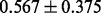	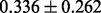	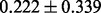

*Note*: We only analyzed protein families with > 5 positive drug-target associations.

We further performed our analysis for different categories of drugs to test whether our approach is more successful for some classes of drugs than for others. For this purpose, we divide drugs into different groups based on their top-level category in the anatomical therapeutic classification (Miller and Britt, 1995) and evaluated each group individually (Supplementary Table S1 and Supplementary Fig. S1).

### 3.3 Example prediction: diclofenac

One example of our method’s predictive power is the identification of similar effects between *PPARg* (MGI:97747) and the drug *diclofenac* (STITCH:000003032). Diclofenac is a non-steroidal anti-inflammatory drug acting primarily as a cyclooxygenase (preferential *COX-2*) inhibitor and is used to treat a variety of acute and chronic pain and inflammatory conditions. In recent years, additional modes of action have been discerned which in many cases have no known mechanism. For example, diclofenac has been shown to inhibit the thromboxane-prostanoid receptor, affect arachidonic acid release and uptake, inhibit lipoxygenase enzymes and activate the nitric oxide-cGMP antinociceptive pathway (Gan, 2010). Using our approach, we have compared the drug effect profile of diclofenac across the gathered phenotypic data and found a high similarity to phenotypes produced by *Pparg* knockout mice. Using our method, 49% of the information content associated with diclofenac’s pharmacological effects can be explained through the hypothesis that it inhibits *Pparg* or its pathway in mice. *Pparg* is a member of the steroid hormone receptor superfamily, which includes the estrogen and thyroid hormone receptors, and regulates the expression of genes involved in inflammation and lipid homeostasis. Despite its anti-inflammatory indications, diclofenac is associated with the induction of dermatitis, alopecia, erythema, exfoliative dermatitis and eczema, along with hepatitis and other widespread systemic phenotypes. Several of these phenotypes can also be identified in mice (Harries and Paus, 2009; Wahli, 2002). In 2002, diclofenac was implicated as a partial agonist of *Pparg*, acting as a competitive antagonist and inhibiting PPARg signaling at normal therapeutic doses (Adamson *et al.*, 2002), suggesting that a significant proportion of diclofenac’s side effects might be explained through this mechanism. The apparent pro-inflammatory effects of diclofenac seen, for example, in the skin are, therefore, likely to be a consequence of the effects on the *Pparg* pathway in non-immune cells, and recent research suggests that it is the effect on the inhibition of PPARg in the pilosebaceceous unit itself that underlies primary cicatricial alopecia, rather than a primary effect on the inflammatory response (Karnik *et al.*, 2009).

We would expect the effects of diclofenac to be concordant with loss of function phenotypes in mutants of its established target, cyclooxygenase 2 (COX-2). A substantial proportion (46%) of the IC associated with the side effects of diclofenac can be explained through COX-2 (*Ptgs2*) knockout phenotypes in mice. For example, the main gastrointestinal effects of diclofenac (inflammation, gastritis, constipation, upper GI tract pain) are consistent with the phenotypes of COX2 knockout mice, as evidenced by the sensitization to inflammatory processes such as induction of colitis, the induction of GI edema and peritonitis seen in COX-2 knockout mice (Morteau *et al.*, 2000).

## 4 DISCUSSION

### 4.1 Choice of semantic similarity measure

The semantic similarity measure we developed for our application has some disadvantages in comparison with symmetric measures, and these are evidenced in the low performance of our approach for low false-positive rates. In particular, for very low false-positive rates, our approach performs worse than random. This lack of performance is a result of our similarity measure, which does not take mismatches between phenotypes into consideration but is based exclusively on *coverage*. A small portion of mouse genes is associated with a large number of phenotypes which almost always cover a large portion of observed drug effects for any drug, and a small portion of drugs is similarly associated with a large number of drug effects in SIDER that cover most observed mouse model phenotypes. For example, the genes *Gt(ROSA)26Sor (gene trap ROSA 26, Philippe Soriano)* (MGI:104735), phosphatase and tensin homolog (Pten, MGI:109583), apolipoprotein E (Apoe MGI:88057) or leptin receptor (Lepr, MGI:104993) are associated with a large number of phenotypes covering most branches of the MP and are ranked among the first mouse genes for most compounds in SIDER. Similarly, drugs such as pregabalin (STITCH:005486971) or fluoxetine (STITCH:000003386) are associated with a large number of drug effects in SIDER and are ranked in the first places for most mouse genes. These artifacts of our similarity measure result in a decreased performance when analyzing the complete dataset and not applying any additional filtering. In particular, the highest-ranking associations resulting from our approach are, in most cases, false positives due to the artifacts generated by the similarity measure, and these artifacts result in a worse-than-random performance in the ROC analysis for low false-positive rates.

However, our measure also has significant advantages over symmetric similarity measures. We have evaluated commonly applied groupwise similarity measures (Pesquita *et al.*, 2009) for our dataset, in particular the Jaccard index and the SimGIC measure. We found the results to be significantly worse than when applying our measure [ROCAUC values are 0.579 for STITCH (mouse) and 0.566 for STITCH (human); raw data available on project Web site]. The major difference between our similarity measure and groupwise measures such as SimGIC is the symmetry property. In particular, symmetric groupwise similarity measures score *mismatches* negatively. In our application, we compare large sets of phenotypes observed as drug effects with the phenotypes observed for single gene deletions in mice. If different drug effects are caused by different proteins with which the drug interacts, we expect only a small portion of the effects to be covered by the phenotypes of a single gene knockout. Negatively scoring all non-matching drug effects introduces noise that increases with the number of drug effects and leads to the significantly lower performance in the ROC analysis. Furthermore, symmetric similarity measures are applicable when comparing essentially similar entities. As most chemical compounds in SIDER interact with many proteins, we compare sets of phenotypes resulting from perturbations of *many* proteins (drug effects) with sets of phenotypes resulting from perturbations of *single* proteins, and in the case of comparing non-similar entities such as drug effects and knockout phenotypes, our non-symmetric similarity measure seems to perform better than symmetric groupwise measures.

### 4.2 Applications in drug repurposing and target discovery

The results of our analyses support the hypothesis that the systematic analysis of similarity between drug effects and mouse model phenotypes can be used to provide insights into drug actions. Although experimental validation is required to determine the suitability of such an approach for the discovery of novel drug targets, our computational evaluation shows that, at least for some protein families, our approach is highly successful (up to 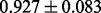 ROCAUC), and therefore may prove promising for integrative approaches toward computational drug repurposing. Our approach is less successful for some protein classes, such as voltage-dependent potassium channel or NAD(P)-binding domain proteins. However, our evaluation datasets contain relatively few positive instances for such classes, indicated by the confidence intervals in Figure 3.

Our work further confirms the hypothesis of Kuhn *et al.* (2013), who mapped 116 mouse phenotype terms to drug effect terms and evaluated 398 knockout mice in an effort to identify proteins that underlie particular drug effects. Kuhn *et al.* formulated the hypothesis ‘that a deletion of a protein in mice is likely to elicit the same phenotype as inhibiting the respective ortholog in humans despite species and methodology differences’. However, while Kuhn *et al.* used this approach in the context of investigating the role of proteins in eliciting drug side effects, our approach provides evidence for the potential of applying mouse models for revealing novel drug-target interactions. Additionally, we systematically evaluated the whole mouse phenome and provide a ranked list of candidate drug targets spanning all drugs in the SIDER database and every protein for which phenotypes have been created in the mouse.

Integrative approaches to drug repurposing and drug target identification take advantage of multiple independent data sources to provide high-confidence predictions of novel indications or novel targets for known drugs (Dudley *et al.*, 2011; Hurle *et al.*, 2013). Our approach will be most useful as a component in integrative approaches to drug repurposing or target identification. In both tasks, the use of animal models is currently largely under-represented despite its potential to provide novel, independent information that strengthens already successful systems (Hurle *et al.*, 2013).

Furthermore, our method is neither based on the ‘guilt-by-association’ principle (Gillis and Pavlidis, 2012) as is applied in side effect-based approaches to drug repurposing (Campillos *et al.*, 2008) or other similarity-based approaches (Gottlieb *et al.*, 2011) nor is our method based on data mining clinical records (Tatonetti *et al.*, 2012); instead, it uses experimental data from genetically modified animal organisms. Our evaluation demonstrates that our method may even be used independently for some categories of targets, particularly steroid hormone receptors, although additional experimental validation is required to further support this hypothesis.

*Conflict of Interest*: none declared.

## Supplementary Material

Supplementary Data
