# Supplementary material for: Mouse model phenotypes provide information about human drug targets
Source: Bioinformatics. 2013 Oct 24;30(5):719–25. doi: 10.1093/bioinformatics/btt613 (PMC3933875; doi:10.1093/bioinformatics/btt613)
Supplement: Supplementary Data [file supp_30_5_719__index.html]

Mouse model phenotypes provide information about human drug targets — Mouse model phenotypes provide information about human drug targets — Mouse model phenotypes provide information about human drug targets — Supplementary Data 

# Mouse model phenotypes provide information about human drug targets

## Supplementary Data

files

**Files in this Data Supplement:**

- Supplementary Data - pdf file
