# Supplementary material for: Mouse model phenotypes provide information about human drug targets
Source: Bioinformatics. 2013 Oct 24;30(5):719–25. doi: 10.1093/bioinformatics/btt613 (PMC3933875; doi:10.1093/bioinformatics/btt613)
Supplement: Supplementary Data [file supp_btt613_drugtargets-supplement.pdf]

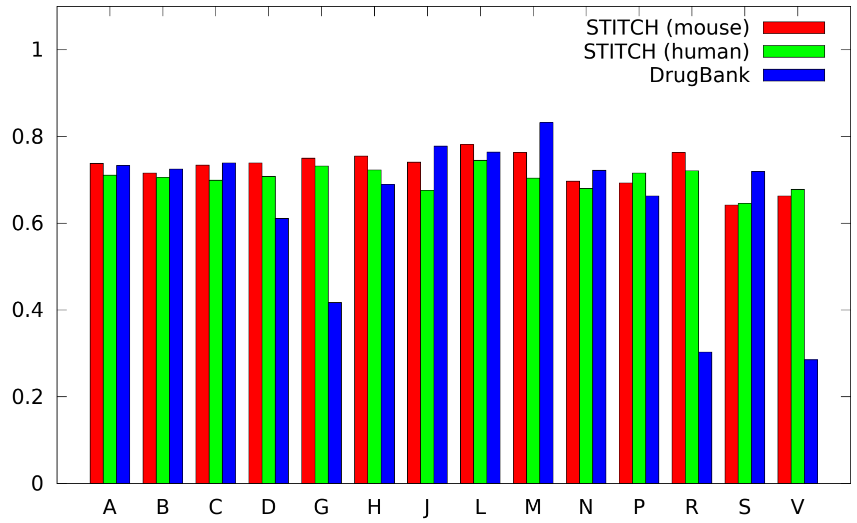

Figure 1: A graphical representation of the ROCAUC values for different drug classes in each of our evaluation datasets. A standards for the ATC class *ALIMENTARY TRACT AND METABOLISM*, B for *BLOOD AND BLOOD FORMING ORGANS*, C for *CARDIOVASCULAR SYSTEM*, D for *DERMATOLOGICALS*, G for *GENITO URINARY SYSTEM AND SEX HORMONES*, H for *SYSTEMIC HORMONAL PREPARATIONS, EXCL. SEX HORMONES AND INSULINS*, J for *ANTIINFECTIVES FOR SYSTEMIC USE*, L for *ANTINEOPLASTIC AND IMMUNOMODULATING AGENTS*, M for *MUSCULO-SKELETAL SYSTEM*, N for *NERVOUS SYSTEM*, P for *ANTIPARASITIC PRODUCTS, INSECTICIDES AND REPELLENTS*, R for *RESPIRATORY SYSTEM*, S for *SENSORY ORGANS*, and V for *VARIOUS*.

**ATC category**	**ROCAUC (STITCH mouse)**	**ROCAUC (STITCH human)**	**ROCAUC (Drug-Bank)**
ALIMENTARY TRACT AND METABOLISM (A)	0.738 (1255)	0.711 (1729)	0.733 (62)
BLOOD AND BLOOD FORMING ORGANS (B)	0.716 (200)	0.705 (286)	0.725 (8)
CARDIOVASCULAR SYSTEM (C)	0.734 (1501)	0.699 (2312)	0.739 (144)
DERMATOLOGICALS (D)	0.739 (518)	0.708 (717)	0.611 (12)
GENITO URINARY SYSTEM AND SEX HORMONES (G)	0.75 (339)	0.732 (488)	0.417 (7)
SYSTEMIC HORMONAL PREPARATIONS, EXCL. SEX HORMONES AND INSULINS (H)	0.755 (51)	0.723 (72)	0.689 (4)
ANTIINFECTIVES FOR SYSTEMIC USE (J)	0.741 (107)	0.675 (217)	0.778 (8)
ANTINEOPLASTIC AND IMMUNOMODULATING AGENTS (L)	0.781 (796)	0.745 (1158)	0.764 (32)
MUSCULO-SKELETAL SYSTEM (M)	0.763 (230)	0.704 (400)	0.832 (42)
NERVOUS SYSTEM (N)	0.697 (1050)	0.68 (2177)	0.722 (139)
ANTIPARASITIC PRODUCTS, INSECTICIDES AND REPELLENTS (P)	0.693 (39)	0.716 (79)	0.663 (6)
RESPIRATORY SYSTEM (R)	0.763 (214)	0.721 (320)	0.303 (11)
SENSORY ORGANS (S)	0.642 (43)	0.645 (104)	0.719 (12)
VARIOUS (V)	0.663 (162)	0.678 (207)	0.285 (5)

Table 1: ROCAUC values and the number of positive instances in the evaluation datasets when dividing our dataset in different drug categories.
